# Efficacy and Mechanism of Moxibustion Treatment on Mild Cognitive Impairment Patients: An fMRI Study Using ALFF

**DOI:** 10.3389/fnmol.2022.852882

**Published:** 2022-05-10

**Authors:** Ziyan Lai, Qingping Zhang, Lingyan Liang, Yichen Wei, Gaoxiong Duan, Wei Mai, Lihua Zhao, Peng Liu, Demao Deng

**Affiliations:** ^1^Department of Radiology, The People’s Hospital of Guangxi Zhuang Autonomous Region, Guangxi Academy of Medical Sciences, Nanning, China; ^2^Department of Acupuncture, The First Affiliated Hospital, Guangxi University of Chinese Medicine, Nanning, China; ^3^Life Science Research Center, School of Life Science and Technology, Xidian University, Xi’an, China

**Keywords:** mild cognitive impairment, dementia, functional magnetic resonance imaging, neurological function, amplitude of low frequency fluctuation

## Abstract

**Background:**

Mild Cognitive Impairment (MCI), as a high risk of Alzheimer’s disease (AD), represents a state of cognitive function between normal aging and dementia. Moxibustion may effectively delay the progression of AD, while there is a lack of studies on the treatments in MCI. This study aimed to evaluate the effect of moxibustion treatment revealed by the amplitude of low-frequency fluctuation (ALFF) in MCI.

**Method:**

We enrolled 30 MCI patients and 30 matched healthy controls (HCs) in this study. We used ALFF to compare the difference between MCI and HCs at baseline and the regulation of spontaneous neural activity in MCI patients by moxibustion. The Mini-Mental State Examination and Montreal Cognitive Assessment scores were used to evaluate cognitive function.

**Results:**

Compared with HCs, the ALFF values significantly decreased in the right temporal poles: middle temporal gyrus (TPOmid), right inferior temporal gyrus, left middle cingulate gyrus, and increased in the left hippocampus, left middle temporal gyrus, right lingual gyrus, and right middle occipital gyrus in MCI patients. After moxibustion treatment, the ALFF values notably increased in the left precuneus, left thalamus, right temporal poles: middle temporal gyrus, right middle frontal gyrus, right inferior temporal gyrus, right putamen, right hippocampus, and right fusiform gyrus, while decreased in the bilateral lingual gyrus in MCI patients. The Mini-Mental State Examination and Montreal Cognitive Assessment scores increased after moxibustion treatment, and the increase in Mini-Mental State Examination score was positively correlated with the increase of ALFF value in the right TPOmid, the right insula, and the left superior temporal gyrus.

**Conclusion:**

Moxibustion treatment might improve the cognitive function of MCI patients by modulating the brain activities within the default mode network, visual network, and subcortical network with a trend of increased ALFF values and functional asymmetry of the hippocampus. These results indicate that moxibustion holds great potential in the treatment of MCI.

## Introduction

Mild Cognitive Impairment (MCI) has evolved over the past two decades to represent a pathological state between normal cognitive aging and dementia (Petersen, 2016). It increases the risk of developing Alzheimer’s disease (AD) or other dementia (Alzheimer’s Disease International M. U., 2021). The prevalence of amyloid pathology, which serves as an essential biomarker of AD, increased from 27% to 71% among MCI patients (Jansen et al., 2015), and the annual incidence rate of individuals with MCI progressing to AD is about 10%–12% (Langa and Levine, 2014). Early and timely effective treatment for individuals in this state may prevent or delay the progression to AD, even revert to normal cognitive function. Unfortunately, there are no approved treatment approaches for MCI.

Moxibustion is a treatment that uses the heat generated by burning moxa sticks to stimulate acupoints, producing a central nervous system regulation similar to acupuncture (Bao et al., 2016). It is a commonly accepted treatment approach for cognitive impairment in East Asia (Aum et al., 2021), with the advantages of painlessness, ease of operation, and fewer adverse events. A systematic review showed that moxibustion could participate in inhibiting oxidative stress and cell apoptosis, adjusting inflammation and Aβ genesis activation of vascular endothelial growth factor, and regulating the metabolic products in the tricarboxylic acid cycle and fatty acid metabolism (Aum et al., 2021). In addition, moxibustion could alleviate the depression-like behavior and adjust tryptophan transport and 5-HT generation (Li et al., 2019). Ha et al. (2020) found that the effect of moxibustion on the cognition of aging mice is associated with the genes and proteins involved in the APP metabolic pathway. Through these mechanisms, moxibustion has good potential in treating cognitive impairment diseases, especially in restraining AD-related Aβ genesis. Recently a review provided credible support for moxibustion in the treatment of AD (A et al., 2021). In the study of vascular dementia rats, it has also been confirmed that moxibustion improves cognitive function, and its mechanism may be related to the inhibition of hippocampal neuronal apoptosis (Yang et al., 2021). The effect of moxibustion on the central nervous system is mainly reflected in the brain function regulation of the default mode network (DMN) including the medial prefrontal cortex and posterior cingulate cortex (Bao et al., 2016). DMN is involved in the process of advanced cognition and is the most susceptible brain network in AD patients (Krajcovicova et al., 2014). Some studies showed that moxibustion could regulate symptoms of MCI (Liu et al., 2020; Zhang et al., 2020), but the efficacy has not been fully validated, and the mechanism remains unclear.

Rest-state functional magnetic resonance imaging (rs-fMRI) has good sensitivity for detecting neuronal activity and functional abnormalities in neurodegenerative diseases. The amplitude of the cerebral blood oxygen level-dependent (BOLD) signals detected by rs-fMRI reflects the energy expenditure and intensity of neuronal activity (Tomasi et al., 2013). The amplitude of low-frequency fluctuation (ALFF) is a data-driven rs-fMRI analysis method that measures the total power of the BOLD signals in the low-frequency range of 0.01 Hz to 0.1 Hz, reflecting abnormal spontaneous neural activity in the brain region (Fox and Raichle, 2007; Zang et al., 2007; Zou et al., 2008). ALFF was able to reflect trends in altered neural activity in AD spectrum disorders and identify differences in healthy people, MCI, and AD (Yang et al., 2018). It was observed to characterize the spontaneous brain activity in MCI or AD patients reliably (Cha et al., 2015). Therefore, we chose ALFF to analyze the abnormal brain function in MCI patients and explore the central mechanism of moxibustion on MCI.

## Materials and Methods

### Participants

A total of 30 MCI patients were recruited through posting advertisements in the local elderly activity center and communities, and 30 normal subjects were recruited as healthy controls (HCs).

The inclusion criteria for MCI patients: (1) between 55 and 75 years old; (2) no dementia; (3) memory impairment was the chief complaint and confirmed by the informant; (4) the ability of daily living was not affected; (5) general cognitive function is basically normal or slightly impaired; (6) without diseases that can lead to the decline of brain function; (7) the clinical dementia rating score: 0.5 points, and the Global deterioration Scale score: 2–3 points.

The exclusion criteria for MCI patients: (1) history of mental illness, such as major depressive disorder; (2) history of neurological diseases that could cause cognitive abnormalities, including encephalitis, brain tumors, and epilepsy; (3) history of other systemic diseases that could cause cognitive abnormalities, including thyroid dysfunction, severe anemia, and syphilis; (4) communication and language impairments, including severe hearing or vision disorders; (5) history of vascular diseases, such as cerebral infarction; (6) taking drugs that could induce cognitive changes or vital organs failure, such as brain, kidneys, and heart; (7) contraindications to MRI; (8) left-handed or two-handed people, or old people with no hands.

### Moxibustion Treatment

According to previous studies on the treatment of cognitive disorders with moxibustion (Aum et al., 2021), we selected six acupoints including Guanyuan, Baihui, bilateral Xuanzhong, and bilateral Zusanli in the MCI group. All MCI patients were treated with moxibustion by the same acupuncturist. Because scarring moxibustion may cause suppuration and leave a scar, our research used non-scarring moxibustion which was similar to other researchers and more acceptable (Pacific WHOROftW, 2007; Bao et al., 2016). When treated with moxibustion, vaseline was applied on the acupoints to protect the subjects’ skin and facilitate the fixing of the moxa-cones. A moxa-cone had a diameter of 1.5 cm, height of 3 cm, and a weight of 5 g. Each acupoint was placed with a moxa-cone, and each moxa-cone was burned for about 3–4 min. The moxa-cone was replaced when the length of the burning moxa-cone was close to the patient’s skin and the patient felt the burning and uncomfortable. Three moxa-cones were used each time in one acupoint. At the end of each duration, the acupuncture points were gently massaged with cotton swabs. The MCI group was treated every other day, with a total of two courses of treatment, 15 times as a course of treatment, and a 3-day rest interval between the two courses.

### Clinical Measurements

The main desired therapeutic effect in the present study is the improvement of cognitive function, which needs to be measured by practical evaluation tools. Currently recognized tools for evaluating cognitive function include the Mini-Mental State Examination (MMSE) and Montreal Cognitive Assessment (MoCA). The higher the MMSE and MoCA scores, the better cognitive function. In our study, MCI patients and HCs were evaluated at baseline, respectively, and then MCI patients were re-evaluated after two months of moxibustion treatment.

### MRI Data Acquisition

All MRI data were collected by a 3.0 T Siemens Magnetom Verio MRI System (Siemens Medical, Erlangen, Germany). A 6-min rs-MRI data was collected at HCs without any intervention. However, MCI patients need to collect MRI data twice totally in without intervention and after a moxibustion treatment. To reduce movement, each subject’s head was immobilized by foam pads in a standard head coil. The 6-min rs-fMRI data were acquired with a single-shot gradient-recalled echo-planar imaging (EPI) sequence with the following parameters: repetition time (TR) = 2,000 ms; echo time (TE) = 30 ms; flip angle (FA) = 90°; field of view (FOV) = 240 mm × 240 mm; matrix size: 64 × 64; slice thickness = 5 mm (no-gap); 31 slices and 180 volumes. High resolution T1-weighted images were then obtained with a magnetization-prepared rapid acquisition gradient-echo sequences (3D MPRAGE) with the following parameters: TR = 1,900 ms; TE = 2.22 ms; FOV = 250 mm × 250 mm; matrix size: 256 × 256; FA = 9°; slice thickness = 1 mm and 176 slices.

### Data Preprocessing

The rs-fMRI data were analyzed by the Data Processing and Analysis for Brain Imaging (DPABI^1^) with Statistical Parametric Mapping, version 12 (SPM12^2^) running under MATLAB platform. The preprocessing steps are as follows: the first five-time points were removed; slice timing correction; head motion correction; normalizing the corrected images spatial to the Montreal Neurological Institute (MNI) space (EPI template with 3 × 3 × 3 mm^3^ voxel size); nuisance signal removal (including head motion parameters, global signal, white matter, cerebrospinal fluid as covariates); linear detrended removal and temporal bandpass filtering (0.01–0.1 Hz). If the translation or rotation of head movement was >2.5 mm or >2.5° in any direction, we excluded subjects’ data from further analysis.

### ALFF Analysis

Each subject used the DPABI program described above for ALFF analysis. The ALFF reflects the intensity of regional spontaneous neural activity and is more sensitive to distinguishing the differences between groups (Fox and Raichle, 2007; Zang et al., 2007; Zou et al., 2008). Fast Fourier transform (FFT) was used to convert the filtered time series to the frequency domain and calculated the square root of the power at each frequency to obtain the amplitude value. ALFF was computed as the sum of the amplitudes of the low low-frequency power range from 0.01 to 0.1 Hz. In order to reduce the overall effect of variability among different participants, each subject used the mean value of within-brain ALFF to standardize ALFF. In addition, the calculation of ALFF was spatially smoothed using a Gaussian kernel with a full width at half maximum of 6 × 6 × 6 mm^3^.

### Statistical Analysis

Demographic and neuropsychological data of the MCI and HCs groups were calculated by SPSS software (version 22.0; IBM, Armonk, New York). Normal distribution continuous variables were tested by two independent samples t-test, and non-normally distributed variables were tested by Mann-Whitney *U*-test. Pearson’s Chi-square test compared categorical variables. In all comparisons, the statistical significance threshold was set at *p* < 0.05.

We used a two-sample t-test to investigate the differences in the normalized ALFF values between MCI patients and HCs (gender, age, and educational level were considered insignificant covariates). Using paired t-tests to quantitatively compare the differences of ALFF values between pre- and post-moxibustion treatment within a gray matter mask using statistical parametric mapping. All statistical graphs are calibrated using the false discovery rate (FDR) correction method, and the significance of the voxel level was set at a *p* < 0.05.

To investigate relationships between changed ALFF of the whole brain and changed clinical symptoms (after treatment − before treatment), and to avoid any unintentional bias induced by region-specific prior hypotheses, a regression analysis was conducted to evaluate the relationships of ALFF values with MMSE in MCI patients. The significance level was set at *p* < 0.05 (FDR correction).

## Results

### Demographic and Clinical Neuropsychological Data

This study included 60 subjects, including 30 MCI patients and matched HCs. There were no statistically significant differences between the two groups regarding age, gender ratio, and educational level. Compared with HCs, the MoCA and MMSE scores were significantly lower (*p* < 0.0001) in MCI patients. Their demographic data and cognitive scores are summarized in Table 1. After moxibustion treatment, the MoCA and the MMSE scores significantly increased in MCI patients (*p* < 0.0001; Table 2).

**Table 1:** Demographic data and cognitive scores in MCI patients and HCs.

Characteristic	MCI (*n* = 30) (mean±SD)	HCs (*n* = 30) (mean±SD)	*p* value
Age (years)	64.30 ± 6.05	66.07 ± 5.91	0.257^a^
Gender (M/F)	10/20	13/17	0.425^b^
Education (years)	11 (11–14)^d^	14 (8–15)^d^	0.129^c^
Pre-MMSE	26 (25–27)^d^	29 (28–30)^d^	0.000^c^
Pre-MoCA	21.83 ± 2.60	25.57 ± 2.06	0.000^a^

*^a^p-values were calculated with a two-sample t-test. ^b^p-values were calculated with a chi-square test. ^c^p-values were calculated with Mann-Whitney U-test. ^d^Median (interquartile range)*.

**Table 2:** Cognitive scores of MCI patients pre- and post-moxibustion treatment.

Characteristic	MCI_POST (*n* = 30)	MCI_PRE (*n* = 30)	*p* value
MMSE change	29.5 (28–30)^d^	26 (25–27)^d^	0.000^e^
MoCA change	27 (25–28.25)^d^	21 (20.75–23.25)^d^	0.000^e^

*MoCA change: post-treatment MoCA score minus pre-treatment MoCA score. MMSE change: post-treatment MMSE score minus pre-treatment MMSE score. ^d^Median (interquartile range). ^e^P-values were calculated with Wilcoxon Signed Ranks Test*.

### ALFF Contrasts

**Figure 1:**
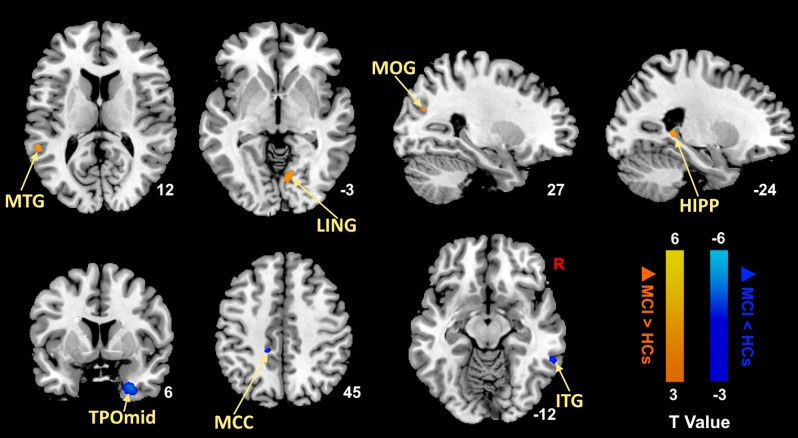
Brain regions with significantly different ALFF values of MCI patients compared with HCs (*p* < 0.05, FDR correction). Warm color represents an increase and cool color represents a decrease respectively. ALFF, amplitude of low-frequency fluctuation; FDR, false discovery rate; MTG, middle temporal gyrus; LING, lingual gyrus; MOG, middle occipital gyrus; HIPP, hippocampus; TPOmid, temporal poles: middle temporal gyrus; MCC, middle cingulate gyrus; ITG, inferior temporal gyrus; MCI, mild cognitive impairment; HCs, healthy controls.

We first compared MCI patients and HCs to find the regions showing altered ALFF values in MCI patients (Figure 1; Table 3). Compared with HCs, MCI patients showed significantly decreased ALFF values in the right temporal poles: middle temporal gyrus (TPOmid), right inferior temporal gyrus (ITG), left middle cingulate gyrus (MCC). As well, the ALFF values in the left hippocampus (HIPP), left middle temporal gyrus (MTG), right lingual gyrus (LING), and right middle occipital gyrus (MOG) increased.

**Table 3:** Brain regions with different ALFF values between MCI patients and HCs in the resting state.

	Brain regions	Brodmann area	Peak MNI coordinates			Cluster size	T-value
			x	y	z		
decreased in MCI	R_TPOmid	36	27	5	−36	78	−4.49
	R_ITG	20	60	−45	−12	107	−3.68
	L_MCC	0	−12	−36	45	102	−3.21
increased in MCI	L_HIPP	37	−24	−39	3	84	3.06
	L_MTG	22	−54	−45	12	86	3.22
	R_LING	18	9	−69	−3	96	3.32
	R_MOG	19	27	−72	24	88	3.02

**Figure 2:**
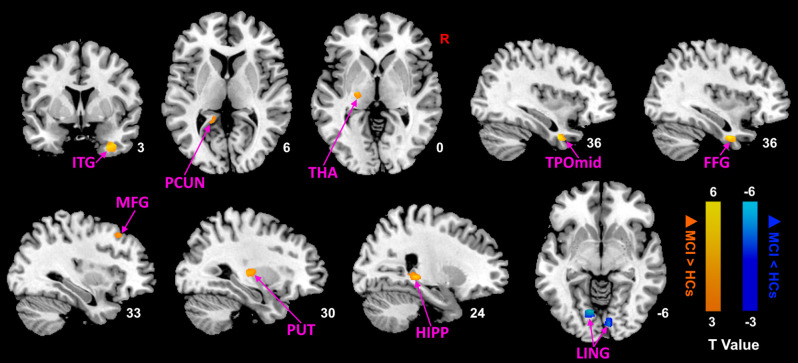
ALFF analysis of MCI patients after moxibustion treatment (*p* < 0.05, FDR correction). Warm color represents an increase and cool color represents a decrease respectively. ALFF, amplitude of low-frequency fluctuation; FDR, false discovery rate; ITG, inferior temporal gyrus; PCUN, precuneus; THA, thalamus; TPOmid, temporal poles: middle temporal gyrus; FFG, fusiform gyrus; MFG, middle frontal gyrus; PUT, putamen; HIPP, hippocampus; LING, lingual gyrus; MCI, mild cognitive impairment; HCs, healthy controls.

Then, we performed comparisons in pre- and post-moxibustion treatment to explore the effects of moxibustion in MCI (Figure 2; Table 4). After moxibustion treatment, the ALFF values in the left precuneus (PCUN), left thalamus (THA), right TPOmid, right middle frontal gyrus (MFG), right ITG, right putamen (PUT), right HIPP, and right fusiform gyrus (FFG) significantly increased. However, the ALFF values in the bilateral LING decreased.

**Table 4:** Brain regions demonstrating altered ALFF values of MCI patients after moxibustion.

	Brain regions	Brodmann area	Peak MNI coordinates			Cluster size	T-value
			x	y	z		
increased after moxibustion	L_PCUN	27	−15	−42	6	105	3.03
	L_THA	0	−21	−18	0	108	3.93
	R_TPOmid	36	36	3	−36	86	5.08
	R_MFG	9	33	24	48	75	3.38
	R_ITG	36	36	3	−39	88	4.8
	R_PUT	48	30	−18	6	108	4.16
	R_HIPP	37	24	−33	0	55	5.26
	R_FFG	36	36	−3	−36	80	6.55
decreased after moxibustion	L_LING	18	−12	−72	−6	88	−5.13
	R_LING	18	9	−84	−6	100	−4.62

### The Association Between ALFF Values and Clinical Changes

**Figure 3:**
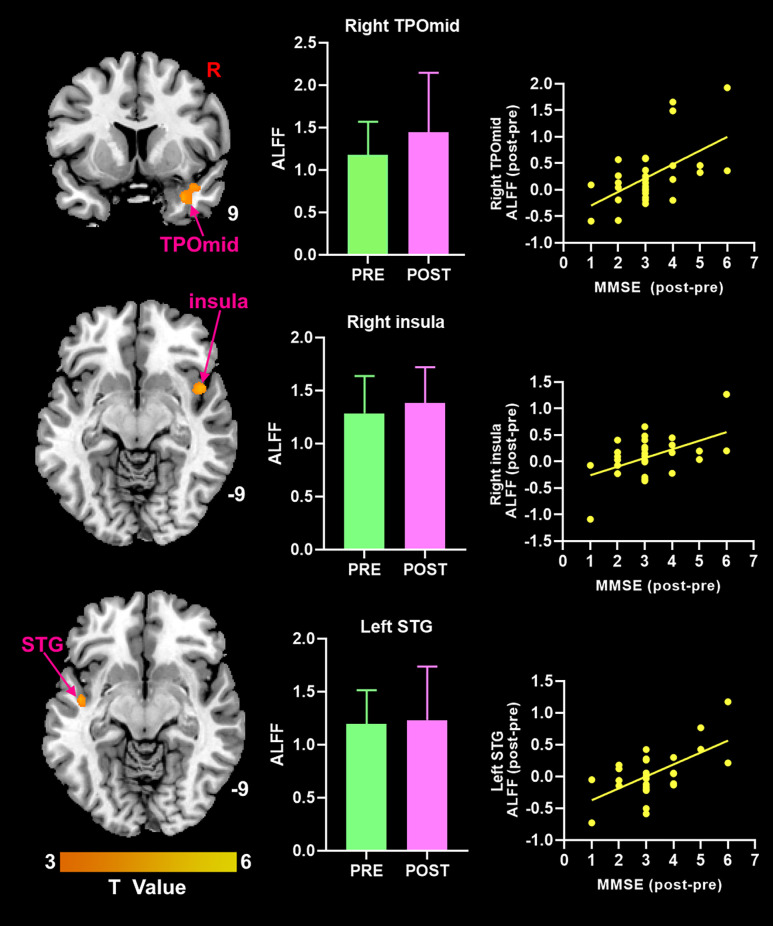
Theincrease of MMSE score was positively correlated with the increase ofALFF value in brain regions (*p* < 0.05, FDR correction). ALFF, amplitude of low-frequency fluctuation; MMSE, Mini-Mental State Examination; TPOmid, temporal poles: middle temporal gyrus; STG, superior temporal gyrus.

After moxibustion treatment, the increase of MMSE score was significantly positively correlated with the increase of ALFF value in the right TPOmid, the right insula, and the left superior temporal gyrus (Figure 3). There was no significant negative correlation between any ALFF value in brain regions in brain regions and MMSE scores in MCI patients.

## Discussion

Our study applied the ALFF approach to fMRI data to investigate the alterations of spontaneous neural activity and the central mechanism of moxibustion in MCI patients. We found that MCI patients showed altered ALFF in a set of brain regions, mainly including the DMN and visual network (VIN). Moxibustion improved the cognitive function by regulating the spontaneous neuronal activity in the DMN, VIN, and subcortical network (SCN) with an increasing trend of the ALFF values. The increase in MMSE score was positively correlated with the increase of ALFF value in the right TPOmid, the right insula, and the left superior temporal gyrus.

### Altered Spontaneous Brain Activity Occurred in MCI Patients

Compared with HCs, the altered ALFF values of MCI patients mainly included in the DMN (the left HIPP, left MTG, and right TPOmid) and the VIN (the right LING, right MOG, right ITG, and left MCC), which were consistent with the results of the previous meta-analysis revealing altered ALFF values in brain regions mainly involving DMN, salience network and VIN (Li et al., 2015; Pan et al., 2017).

The DMN, as the most extensively studied brain network among AD patients and high-risk subjects, is related to advanced cognitive functions (Krajcovicova et al., 2014). This network is believed to involve self-referencing processes, intuitive thinking, memory processes, and perhaps information integration and preservation (Beason-Held et al., 2017). The cortical regions of DMN distribution widely overlapped with areas of early Aβ plaque deposition (Hoenig et al., 2018; Zott et al., 2018). Huijbers et al. (2015) mentioned that amyloid-β positive patients with MCI showed increased hippocampal activation and Clinical Dementia Rating score. Yang et al. (2018) observed increased ALFF value in the left HIPP, and spontaneous activity of neurons in this region enhanced to maintain cognitive ability. Some of the increased connectivity of DMN can compensate for interruptions in other areas of the network (Beason-Held et al., 2017). It suggests that the brain region with a reduced ALFF value represents an impaired function, while the increased region may represent a functional impairment or a compensatory mechanism. In other words, we could call it the perturbed activity pattern of the DMN. The abnormality of DMN may be an important feature of the neuroimaging model of MCI patients, which can provide an auxiliary MRI basis for the clinical diagnosis of MCI.

In addition to the DMN, the brain regions of Tau pathological accumulation and the VIN distribution also have a high overlap (Hoenig et al., 2018). The occipital gyrus and LING are steady in the visual system network (Yang et al., 2015). Metabolomics and transcriptomics studies confirmed that the metabolite changes of ITG are related to the severity of AD pathology (Mahajan et al., 2020). Visual processing difficulties are often present in AD and MCI (Krajcovicova et al., 2017). In the Parkinson’s study, the stability of the VIN and DMN are associated with the severity of visual hallucinations symptoms (Dujardin et al., 2020). It may be inferred that visual hallucinations also occur in MCI patients with the same altered VIN spontaneous neural activity. Meanwhile, inefficient VIN activation might interfere with higher cognitive processing in AD patients (Li et al., 2015). Consistent with the previous conclusions, we conclude that VIN also has a functional impairment and a compensation mechanism. Furthermore, there may be some correlation in terms of vision between VIN and DMN wait for verification.

### Moxibustion Modulates the Brain Region of MCI Patients

Our study found extensive changes in ALFF values in the brain region mainly involving the DMN, VIN, and SCN after moxibustion in MCI patients, and the ALFF values primarily increased. Meanwhile, the MMSE and MoCA scores increased after moxibustion, indicating the improvement of cognitive function. The perturbed activity pattern of DMN is a crucial feature of abnormal central nervous activity in MCI and AD. Our study revealed that the target brain regions of moxibustion are mainly located in DMN, VIN, and SCN. Moreover, the ALFF value in the right TPOmid of MCI patients was decreased but increased after moxibustion and was positively correlated with the MMSE score. Our results indicate that DMN is a vital target region of moxibustion’s central nervous regulation mechanism, consistent with the previous study (Bao et al., 2016). In particular, the changed ALFF values in the right ITG, right TPOmid, and the right LING were reversed, from decreasing to increasing, and the abnormalities of the HIPP happened on the contralateral side. These results provide evidence that moxibustion can regulate central nervous spontaneous activity and improve cognitive function in MCI patients.

The role of DMN and VIN in AD-related diseases has been discussed earlier in this article. In addition, this study also increased ALFF values in SCN, including THA and PUT. They are part of the corticobasal ganglia–thalamic circuits that are critical for a variety of cognitive functions, like working memory and perceptual decision-making (Wei and Wang, 2016). In comparison between dementia-related processes and aging, authors found that HIPP and amygdala demonstrated high disease-related effects, while the THA, PUT and white matter revealed weakly related to cognitive decline but a strong association with aging (Wachinger et al., 2016). Functional neuroimaging and pathological studies found that the THA and PUT activated in the process of reading and speaking (Seghier and Price, 2010). The THA is associated with the transmission and integration of cognitive information; its medial part includes the Papez circuit, which is involved in memory and learning processes (Beh et al., 2013). A previous study presented that with cognitive decline in AD, the integrity of thalamic connections is gradually compromised (Zhu et al., 2015). The PUT is traditionally associated with reinforcement learning and motor control, including speech articulation (Viñas-Guasch and Wu, 2017). The ALFF values in the right PUT were significantly related to the memory decline over time and more severe AD pathology (Ren et al., 2016), and the hypoactivation of PUT was found in task-based fMRI studies in MCI (Li et al., 2015). In the present study, altered ALFF values in the THA and PUT were only found after treatment, and we speculate that they may be related to normal aging and affected by the abnormal activities of other brain regions before treatment.

HIPP is a crucial part of DMN and plays a vital role in the brain’s ability for memory storage and retrieval (particularly episodic memories in humans; Knierim, 2015) and social behavior adaptation (Montagrin et al., 2018). Hippocampal atrophy is one of the most readily available and effective biomarkers of AD (de Flores et al., 2015), and the structural asymmetry of HIPP, amygdala, caudate, and cortex may be a sensitive biomarker to predict the progression from MCI to AD (Wachinger et al., 2016). Furthermore, the functional asymmetry of the HIPP is critical for cognitive processes, and it is found to increase in dementia (Muntsant and Giménez-Llort, 2020). The study of neurofunctional topography of the human HIPP segmented the left HIPP into emotional processing, cognitive operations, and post perceptual cluster, while the segmentation of the right HIPP required further confirmation (Robinson et al., 2015). Neurodegeneration associated with aging and disease may preferentially affect the left hemisphere, which innervates speech and movement (Minkova et al., 2017). In AD spectrum disease, the asymmetric pattern of HIPP was found a gradual trend of HC-SCD-MCI. A meta-analysis showed increased ALFFs in the left HIPP in amnestic MCI (Pan et al., 2017). Our results of increased ALFF values in the left HIPP in MCI compared with HCs were in line with the literature. After moxibustion, the spontaneous brain functional activity in right HIPP increased compared with baseline status. In the research of spatial navigation and episodic memory, the activation of the right HIPP prompts the heterocentric spatial model (Iglói et al., 2010). Disruption of the right hippocampal structure has been found in the subcortical vascular MCI (Lyu et al., 2019), and the increased susceptibility of iron deposition in the right HIPP of subcortical vascular MCI patients is related to memory and language functional tests scores (Sun et al., 2017). More importantly, abnormalities in the right HIPP are associated with MMSE and MoCA scores in SCD which is proposed as a high-risk state of AD (Yue et al., 2018). Although the ALFF value of the right HIPP was not statistically correlated with the cognitive scores of the patients, the cognitive scores and function of MCI patients significantly improved after moxibustion. Thus, moxibustion might improve cognitive function by modulating the functional activity of the right HIPP. Previous studies have also shown that moxibustion improves cognitive function, and its mechanism may be related to the inhibition of hippocampal neuron apoptosis and Aβ deposition which might be the mechanism of inducing AD (Aum et al., 2021; Yang et al., 2021). It is concluded that moxibustion might be of great potential in the treatment of MCI.

It is worth noting that the present study showed a gender imbalance in the random sample of MCI among women as twice compared to men, which is consistent with the recent comprehensive study on the prevalence, risk factors, and management of dementia and MCI in China (Jia et al., 2020). Although gender differences in the incidence of AD are controversial across studies, most results also support a higher prevalence in women (Mielke et al., 2014; Fiest et al., 2016; Nebel et al., 2018). The possible mechanisms of gender differences in MCI and AD mainly include differences in genes, sex hormones, brain structure, glial cells, and immunity (Fisher et al., 2018; Honarpisheh and McCullough, 2019), while MRI has confirmed gender differences in brain structure. Studies using longitudinal MRI data from AD to MCI also confirmed different patterns of gray matter atrophy in different genders, especially in bilateral anterior cuneus nucleus, caudate nucleus, THA, and MTG (Skup et al., 2011). In addition, women with MCI lose more volume in the HIPP than men (Sundermann et al., 2017). Current studies on precision medicine suggest the need to pay attention to gender in the clinical manifestations, pathophysiological mechanisms, and treatment of patients with MCI or AD (Nebel et al., 2018). It may be of interest to have a sex-disaggregated study of brain fMRI in MCI patients in the future with a large sample size.

In conclusion, there existed changes in ALFF values of brain regions related to cognition in MCI, mainly involved in the DMN and VIN. Moxibustion might improve the cognitive function of MCI patients by modulating the brain activities in regions involving the cognitive processes with a trend of increased ALFF values, as well as the functional asymmetry of the HIPP. In particular, DMN is a vital target region of moxibustion’s central nervous regulation mechanism.

This study used the ALFF method to study the abnormal brain activity of MCI patients and the regulation of moxibustion on the brain activity of MCI patients, but this study had limitations. First of all, our samples used a cross-sectional design, and a longitudinal study needs further exploration. Second, we only used ALFF as the analytical method instead of more research methods to verify together, such as regional homogeneity and functional connectivity. The role of moxibustion in the regulation of MCI patients needs the exploration of multimodal brain imaging technology. Third, physiological noise during scanning, such as breathing movement, might affect the stability of fMRI signals. Much spurious information might be arisen due to the low-frequency spontaneous fluctuation of BOLD signals and the physiological noise such as cardiac and respiratory cycles (Li et al., 2014). Fourth, our study’s relatively small sample size may have influenced the results, and future studies with a larger sample size will help provide the credibility of our results. Finally, as the entire course of moxibustion lasted for two months, challenges were presented for healthy controls. Therefore, we did not use moxibustion to intervene HCs.

## Data Availability Statement

The raw data supporting the conclusions of this article will be made available by the authors, without undue reservation.

## Ethics Statement

The studies involving human participants were reviewed and approved by The Medicine Ethics Committee of First Affiliated Hospital, Guangxi University of Chinese Medicine. The patients/participants provided their written informed consent to participate in this study.

## Author Contributions

DD contributed design of the study and revised the final version of the manuscript. ZL, QZ, and YW conducted data collection, researched and drafted manuscripts. PL, LL, and GD were involved in manuscript editing. PL, WM, and LZ contributed to MRI processing and data analysis. All authors contributed to the article and approved the submitted version.

## Conflict of Interest

The authors declare that the research was conducted in the absence of any commercial or financial relationships that could be construed as a potential conflict of interest.

## Publisher’s Note

All claims expressed in this article are solely those of the authors and do not necessarily represent those of their affiliated organizations, or those of the publisher, the editors and the reviewers. Any product that may be evaluated in this article, or claim that may be made by its manufacturer, is not guaranteed or endorsed by the publisher.

## Footnotes

^1^ http://rfmri.org/dpabi

^2^ http://www.fil.ion.ucl.ac.uk/spm
